# Sliced Lotus Root as a Hypopharyngeal Foreign Body

**DOI:** 10.7759/cureus.19296

**Published:** 2021-11-05

**Authors:** Masahito Katsuki

**Affiliations:** 1 Neurosurgery, Itoigawa General Hospital, Itoigawa, JPN

**Keywords:** lotus root, foreign body, airway, hypersalivation, sore throat

## Abstract

Pharyngeal foreign bodies are medical emergencies that require airway protection. Rapid diagnosis and adequate treatment are needed. However, in elderly patients with dementia, diagnosing foreign body aspiration sometimes seems difficult only from the medical history, so we should maintain a high degree of suspicion when treating patients with unexplained symptoms. We herein present a 95-year-old woman with hypersalivation and sore throat two hours after dinner. Due to her mild dementia, sufficient medical history could not be obtained. She could walk and talk, but could not swallow. Her vital signs were all within normal limits. There were no abnormal findings in the oral cavity. The neck x-ray images revealed the patent airway and foreign body in the hypopharynx. We used Macintosh laryngoscope and Magill forceps to remove the foreign body, which was a sliced lotus root with a diameter of 61 mm. After removal, she could swallow, and her symptoms rapidly improved. We should suspect this if the patient presents unexplained pharyngeal symptoms.

## Introduction

Death due to foreign body aspiration is common in infants and the elderly [1], and pharyngeal foreign bodies are medical emergencies that require airway protection [2]. Therefore, rapid diagnosis and adequate treatment are needed. However, in elderly patients with dementia, diagnosing foreign body aspiration only from the medical history is sometimes difficult, so we should maintain a high degree of suspicion when treating patients with unexplained symptoms [3]. We report the case of a woman with hypersalivation and sore throat after dinner. She had a sliced lotus root in the hypopharynx.

## Case presentation

A 95-year-old woman presented with hypersalivation and sore throat two hours after dinner. She had mild dementia and lived with her family with their support but could eat without any help. She did not have any allergies. She had hypertension and took two types of antihypertensive drugs. Due to her mild dementia, we could not obtain a sufficient medical history. She could walk and talk, but she could not swallow. Her vital signs were all within normal limits. There were no abnormal findings in the oral cavity. As a differential diagnosis, allergic diseases rose, and we took neck x-ray images to evaluate the airway patency.

**Figure 1:**
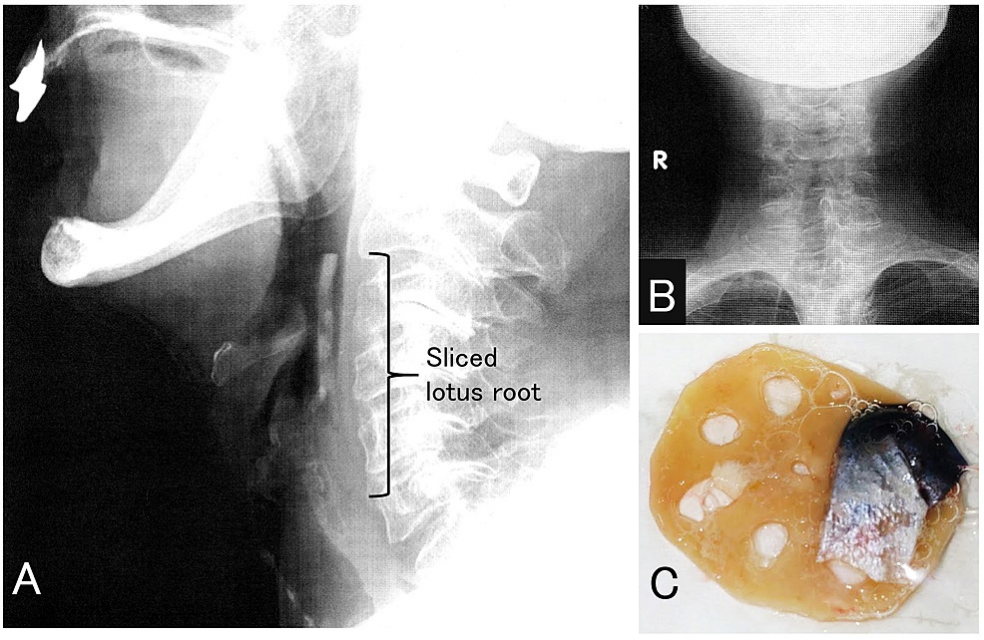
X-ray images and removed lotus root A: Side view of the neck (x-ray). The sliced lotus root was present in the hypopharynx, and it wedged into the proximal part of the esophagus. B: Frontal view of the neck (x-ray). C: The removed lotus root and fish skin. The diameter and thickness of the lotus root were 61 mm and 4 mm, respectively.

The neck x-ray images revealed the patent airway and foreign body in the hypopharynx. It wedged into the proximal part of the esophagus (Figure 1A, 1B). We used a Macintosh laryngoscope with blade #3 and Magill forceps to remove the foreign body without any anesthesia, which was a sliced lotus root. After removal, she could swallow, and her symptoms rapidly improved. The removed lotus root was with fish skin. Its diameter and thickness were 61 mm and 4 mm (Figure 1C).

## Discussion

We presented the case of a 95-year-old woman with a foreign body in the hypopharynx. Her medical history and symptoms are not specific to a pharyngeal foreign body, but we should suspect it if the patient presents unexplained pharyngeal symptoms. Therefore, we should perform a radiological examination for such patients.

The presence of foreign bodies in the airways can be life-threatening. Bronchoscopy, both flexible and rigid, has become the gold standard for diagnosing and treating patients with suspected airway foreign bodies. Although otolaryngologists and thoracic surgeons have traditionally treated foreign bodies in the airways, the development of smaller diameter flexible bronchoscopes has enlarged the involvement of physicians or non-specialists in otolaryngology and thoracic surgery in the diagnosis and therapy of patients with foreign bodies in the airway.

According to data from the National Security Council, around 80% of occurrences occur in children under the age of 15, with the remaining 20% occurring in individuals above 15 years of age. With approximately 7000 fatal foreign body aspiration incidents documented in 2019, it is the fourth greatest cause of unintentional home and community mortality in the United States. Foreign body aspiration causes the most deaths in children under the age of one year. and the elderly over 75 years of age. For those who are over 75 years old, the frequency climbs, with the fatalities peaking at 85 years old [4]. A similar trend with regard to the elderly and foreign body aspiration was confirmed in Japan [5]. This is because the swallowing function of people deteriorates with age and older people are more likely to choke on food [6]. However, the incidence rate of foreign body aspiration is as low as 0.66 per 100,000 [7]. Therefore, due to its rarity, we need to be prepared for likely fatal cases. Our patient also had no obvious episode of aspiration, which could have been missed.

Risk factors for foreign body aspiration include loss of consciousness, alcohol intoxication, anesthesia, swallowing dysfunction due to age, other comorbidities, and medication [8]. Our patient was elderly and had dementia, so the risk was high.

A large study in Japan regarding fatal foreign body aspiration revealed that the number of deaths due to food suffocation was highest on New Year’s Day, frequently occurred at home, and was highest among people aged over 75 years. Approximately 4,000 food choking deaths occurred in Japan yearly from 2006 to 2016 [5]. The seasonal variations, such as Japan's high death rate in January, were not detected in a United Kingdom study [9], and this could be due to the Japanese New Year practice of eating rice cake. To prevent this, mass media and the government have issued warnings about the fatal choking risk due to rice cake and the public has become more aware. This has led to a decrease in such incidence among people over 75 years old [5]. However, in our case, a lotus root was found as the foreign body instead of rice cake. Boiled or fried lotus root is widely eaten in Japan and China. It has a crunchy texture, so we often cook it as slices, not cut into small pieces. In our case, the time from the onset to the consultation was short, i.e., two hours, so the lotus root was rigid. We could thus remove it by Macintosh laryngoscope and Magill forceps. However, if more time had passed, the lotus roop would have become soft [3]. Forceps may increase the likelihood of foreign body fragmentation and further distal wedging, which are less accessible to extraction [1]. Therefore, the removal procedure should be carefully chosen discussing with gastroenterologists and otolaryngologists.

## Conclusions

We presented a case of a 95-year-old woman with a foreign body in the hypopharynx. The foreign body was sliced lotus root, which was not so common. Her medical history and symptoms were not specific to a pharyngeal foreign body, but we should suspect it if the patient presents unexplained pharyngeal symptoms. If we find a foreign body in the pharynx, the removal procedure should be discussed with gastroenterologists and otolaryngologists. Further investigation on foreign body aspiration is needed to clarify what food causes it in Japan.
